# Interleukin-6 May Contribute to Mortality in Parkinson's Disease Patients: A 4-Year Prospective Study

**DOI:** 10.1155/2015/898192

**Published:** 2015-08-16

**Authors:** Michal Dufek, Irena Rektorova, Vojtech Thon, Jindrich Lokaj, Ivan Rektor

**Affiliations:** ^1^First Department of Neurology, Masaryk University, St. Anne's University Hospital, 656 91 Brno, Czech Republic; ^2^Central European Institute of Technology (CEITEC), Brain and Mind Research Programme, 625 00 Brno, Czech Republic; ^3^Department of Clinical Immunology and Allergy, Faculty of Medicine, Masaryk University, St. Anne's University Hospital, 656 91 Brno, Czech Republic

## Abstract

*Objectives*. The association between abnormal serum immunomarkers and mortality in 53 consecutive Parkinson's disease patients was studied. *Materials and Methods*. The plasma level of specific inflammatory cytokines was investigated: mannan-binding lectin (MBL), interleukin- (IL-) 6, and tumor necrosis factor-alpha (TNF-*α*). The baseline serum immunomarkers obtained from patients who died (*n* = 16) during a four-year follow-up period were compared with the data of patients who survived (*n* = 37). *Results*. The baseline level of IL-6 was significantly higher in the deceased patients than in the survivors. Elevated IL-6 levels and age were major independent contributors to disease mortality. Differences between other plasma cytokine level abnormalities were not significant. *Conclusion*. This study showed that IL-6 elevation may be a marker of increased mortality risk in Parkinson's disease patients. The inflammation may act in association with other factors and comorbidities in progressive neurodegenerative pathology.

## 1. Introduction

Aging is associated with a low-grade increase of inflammatory activity. This increase is usually explained by the influence of a wide range of environmental factors, including smoking, obesity, infections, genetic factors, the declining functions of sex hormones, and age-related comorbidity. Peripheral blood monocytes have an increased capacity to produce proinflammatory cytokines in the elderly. Some authors [1–3] described elevated IL-6, TNF-*α*, C-reactive protein (CRP), or fibrinogen in the serum of elderly people. IL-6, CRP [4–8], and TNF-*α* (9) are associated with higher mortality in the elderly. Parkinson's disease (PD) typically occurs in the sixth or seventh decade of life. Neuroinflammatory processes participate in PD pathogenesis. Microglia in the substantia nigra produce proinflammatory cytokines, that is, IL-6, IL-1*β*, and TNF-*α*, and their concentrations are increased in the striatum [9] and in cerebrospinal fluid (CSF) [10–12]. There are a few reports about plasma elevations of TNF-*α* and IL-6 in PD patients [13–15]. Microglia-mediated neuroinflammation is thought to play an important role in the pathogenesis of PD, but it is not clear whether the occurrence of the inflammatory markers in PD represents a causal factor or a consequence of the ongoing neurodegenerative process [16–18].

We recently reported changes in the spectrum of serum immunomarkers (primarily TNF-*α*, MBL) in patients with PD but found no clear correlation between cytokine abnormality and the severity of the motor and psychological aspects of PD [15]. In the current study we hypothesized that abnormal proinflammatory cytokine levels (TNF-*α* and IL-6) may indicate the presence of a cerebral inflammatory process that might contribute to fatal outcomes in PD patients. And if so, are the changes in these 2 plasma cytokines an age-related factor or an age-independent contributor to mortality? We choose TNF-*α* for its potentials to induce acute inflammation and to mediate the systemic effects of inflammation and IL-6 for its unique role as the major determinant of lymphocytes differentiation, including both B and T lymphocytes, as well as the most important inducer of hepatocyte synthesis of acute-phase proteins and also as a terminator of upregulatory inflammatory cascade and inhibitor of IL-1 and TNF-*α* synthesis via IL-1 receptor antagonist.

We extended therefore the baseline cohort (from 29 in the first study to 53) and performed a follow-up analysis of our cohort after four years. The baseline plasma cytokine levels of TNF-*α* and IL-6 obtained from patients who survived were compared to the data of those who died. An increase of the plasma level of IL-6 and TNF-*α* in PD as well as in mortality studies of elderly population was repeatedly reported in the literature [4, 7, 14]; the TNF-*α* level was elevated also in our previous study [15].

No data about an association between mortality and inflammatory markers in PD have been previously published to our knowledge.

## 2. Subjects and Methods

We examined 53 consecutive patients from the Movement Disorder Center in Brno (38 men and 15 women) ages 55–85 (mean 68.66 ± 6.79). None of the patients had vascular parkinsonism or was significantly impaired by another comorbidity. The ethics committee (The St. Anne's University Hospital Ethic Committee) approved the study and informed consent was obtained from each patient. The patient cohort was defined by the investigation of the inflammatory biomarkers. This cohort is part of a larger well-described cohort that was studied with respect to vascular factors and published elsewhere [19, 20].

A baseline examination of the immunomarkers (IL-6, TNF-*α*, C-reactive protein, serum amyloid A, alpha 1-antitrypsin, orosomucoid, ceruloplasmin, alpha 2-macroglobulin, transferrin, prealbumin, MBL) and factors of the complement system (C1q, C1-INH, C3, and C4) in serum was performed on 53 patients in 2005. IL-6 and TNF-*α* were chosen for the follow-up study as they act as possible proinflammatory factors.

All 53 patients were invited for a follow-up evaluation, four years after the baseline visit. Of those, 27 patients attended the follow-up visit, 16 had died, and the other 10 patients were either unmotivated to come for examination or bedridden in an advanced stage of the disease.

Patient outcome at four years after the baseline examination was determined by assessing the reports of movement disorders specialists, general practitioners, and caregivers and from the death register.

The most frequent cause of death (in eight patients) was “generalized atherosclerosis” according to the death certificates which was most probably a diagnosis that reflected and covered the end stage of PD. Pneumonia or another pulmonal disease was reported in three subjects, ischemic heart disease in one subject, femoral phlebitis in one subject, and ischemic stroke in one patient. In three cases, the data were unreliable.

In our cohort, one patient died from a stroke, one from a myocardial infarction, one from lung cancer, two from bronchopneumonia, one from an accident, one from complications after femur coli fracture, one from a liver dysfunction, and eight from end-stage generalized atherosclerosis.

The serum immunomarkers levels of the deceased patients (Group 1, *n* = 16) were compared to the data of surviving patients (Group 2, *n* = 37).

An independent statistician evaluated all the data.

The nonparametric Mann-Whitney *U* test for independent variables was used to test differences in age, disease duration, and serum immunomarkers levels between Group 1 and Group 2. To confirm the hypothesis of the independence of age and abnormal serum immunomarkers, the acquired data were tested using the general regression method (GRM) with a post hoc analysis of variance (ANOVA). IL-6 values were used as the dependent variable, age as a continuous independent variable, and the parameter “survivors-nonsurvivors” as a categorized variable.

According to the statistician the testing of defined hypothesis ((1) the changes in plasma cytokine levels markers may be markers of increased mortality in PD and (2) if so the changes in plasma cytokine levels an age-related factor or an age-independent contributor to mortality) does not require correction for multiple comparison.

## 3. Results

At the baseline examination, major serum immunomarkers abnormalities were observed. The TNF-*α* was increased in 15 patients (28.3%); IL-6 was increased in 7 patients (13.2%). Only minor changes were detected in other immunomarkers.

Statistically significant differences between Group 1 and Group 2 are shown in Table 1.

The data of Group 1 and Group 2 differed significantly (*p* < 0.05) in the IL-6 (*p* = 0.034) level and age (*p* = 0.000) and almost significantly in TNF-*α* (*p* = 0.058); the data did not differ with regard to disease duration.

Correlation analysis showed no significant correlation between age and IL-6 in Group 1 (*r* = −0.014, *p* = 0.958) and Group 2 (*r* = −0.108, *p* = 0.549); that is, age was not associated with IL-6 levels in either group.

This result was further proven by the GRM analysis (multiple regression coefficient *R* = 0.462, *p* = 0.004).

Analysis of variance (ANOVA) revealed that IL-6 did not depend on age (*p* = 0.729, *F* = 0.121, *df* = 1) but on the “survivors-nonsurvivors” parameter (*p* = 0.001, *F* = 11.560, *df* = 1).

## 4. Discussion

Disease-related degenerative brain changes and coincident diseases are primary causes of death in PD [21].

There is a link between elevated proinflammatory cytokines and mortality. Aging is associated with increased circulating levels of proinflammatory cytokines. Bruunsgaard et al. described an association between IL-6 elevation and death for both sexes in people 80 years old [7]. Harris et al. [4] showed a link between elevated IL-6, CRP, and mortality in healthy nondisabled seniors over 65 years old. Some studies reported an association between mortality and elevated IL-6 in hemodialysis patients [6] and in patients with multiple injuries [5]. IL-6 is a multifunctional cytokine with important regulatory roles in immune processes, the metabolism of fat, proteins, and carbohydrates, and the induction of procoagulant changes [22, 23].

In our cohort, both age and IL-6 were independent contributors to mortality in PD patients. Our study results did not demonstrate a correlation between IL-6 levels and age which could have been caused by a relatively small cohort study. Our findings support the hypothesis that inflammation contributes to the death rate in PD patients. Various underlying pathological processes may contribute to the increased level of IL-6. One process may be the impairment of brain vessels. Atherosclerosis is associated with low-grade inflammation [24–27]. Vascular mechanisms may also contribute to mortality in PD [20, 21, 28, 29] and cardiocerebrovascular disease was the cause of death in the majority of our deceased patients.

Proinflammatory cytokines can migrate between systemic circulation and brain in both directions which could explain the comorbidity of systemic illnesses with neurological disorders. There are three pathways for the transport of proinflammatory cytokines from systemic circulation to brain: cellular, humoral, and neural [30]. Moreover, DAMPs can prime glial cells to express proinflammatory cytokines, including IL-6. When expressed, these cytokines activate granulocytes, monocytes/macrophages, NK cells, and T cells and together contribute to the pathophysiology of neuroinflammation. Chronic neuroinflammation may deepen a vicious circle [31].

We have published that the decedents were older, with more severe clinical cognitive and vascular impairment than the survivors in our cohort [20]. Some studies reported that older age, longer disease duration, cognitive impairment, and hallucinations were strongly associated with greater mortality risk [32, 33]. In our cohort, despite similar disease duration, the deceased patients had a more advanced stage of parkinsonism at the baseline visit, suggesting that these patients may have had a more rapidly progressing disease. Future studies should address the question of whether inflammation contributes to or reflects the rate of PD progression.

We have to point out several study limitations. The cohort of examined patients was rather small in size. There is an inherent bias in comparing patients who died to those who did not, as the former group could have had more associated conditions. None of the PD patients was significantly impaired due to comorbidities at the baseline assessment but we lack accurate data concerning comorbidities in the period preceding death in those who died. We also lack the clinicopathological correlation. One could argue that a peripheral concentration of cytokines may not precisely reflect inflammation in the central nervous system since cytokines may be of other than CNS origin. But we assume that this is less probable as the increase of IL-6 specifically in PD has been reported in postmortem brain studies [34] and in vivo in CSF [10], as well as in blood [14, 15].

In summary, this study showed that elevated IL-6 may be a marker of increased mortality risk in PD patients, but based on our study design and current data it is difficult to conclude that increase of mortality in PD is due to an overall inflammatory phenomenon or even due to central inflammation. The present results cannot contribute to the debate whether elevated cytokines is a causal factor or a response to neurodegeneration, but it may indicate that inflammation has clinical relevance in PD. The inflammation may act in association with other factors and comorbidities on the terrain of the progressive neurodegenerative pathology. Larger prospective longitudinal and repeated-in-time studies are warranted in at-risk PD populations and in diagnosed PD cohorts in order to elucidate the exact role of plasma cytokines in brain inflammation as a potential etiologic and prognostic contributor to PD development and progression and specifically to mortality.

## Figures and Tables

**Table 1:** Characteristics of the investigated patients and the laboratory results in Groups 1 and 2.

	Group 1					Group 2					*p* level
	Valid *N*	Median	Lower Q	Upper Q	Range	Valid *N*	Median	Lower Q	Upper Q	Range	
Age at baseline (years)	16	74.1	72.0	75.5	28.2	37	64.2	63.0	69.1	29.0	**0.000**
Diagnosis (years)	16	9.0	6.5	16.1	22.1	37	8.0	5.1	10.0	18.1	0.150
IL-6 (pg/mL)	16	2.71	1.92	4.51	4.72	37	1.93	1.90	2.31	4.11	**0.035**
TNF-*α* (pg/mL)	16	7.21	5.95	10.15	24.40	37	5.81	4.42	8.90	14.72	0.058

IL-6 = interleukin-6.

TNF-*α* = tumor necrosis factor-alpha.

*p* = nonparametric Mann-Whitney *U* test for independent components.

Significant differences are in bold.
